# Obesogenic dietary intake in families with 1-year-old infants at high and low obesity risk based on parental weight status: baseline data from a longitudinal intervention (Early STOPP)

**DOI:** 10.1007/s00394-015-0899-9

**Published:** 2015-04-18

**Authors:** Viktoria Svensson, Tanja Sobko, Anna Ek, Michaela Forssén, Kerstin Ekbom, Elin Johansson, Paulina Nowicka, Maria Westerståhl, Ulf Riserus, Claude Marcus

**Affiliations:** Division of Pediatrics, Department of Clinical Science, Intervention and Technology, Karolinska Institutet, Karolinska University Hospital Huddinge B62, 141 86 Stockholm, Sweden; Institute of Human Performance, University of Hong Kong, Hong Kong, China; Division of Clinical Physiology, Department of Laboratory Medicine, Karolinska Institutet, Stockholm, Sweden; Clinical Nutrition and Metabolism, Department of Public Health and Caring Sciences, Uppsala University, Uppsala, Sweden

**Keywords:** Dietary intake, Food intake, Infant, Parents, Obesity, Infant feeding

## Abstract

**Purpose:**

To compare dietary intake in 1-year-old infants and their parents between families with high and low obesity risk, and to explore associations between infant dietary intake and relative weight.

**Methods:**

Baseline analyses of 1-year-old infants (*n* = 193) and their parents participating in a longitudinal obesity intervention (Early STOPP) were carried out. Dietary intake and diet quality indicators were compared between high- and low-risk families, where obesity risk was based on parental weight status. The odds for high diet quality in relation to parental diet quality were determined. Associations between measured infant relative weight and dietary intake were examined adjusting for obesity risk, socio-demographics, and infant feeding.

**Results:**

Infant dietary intake did not differ between high- and low-risk families. The parents in high-risk families consumed soft drinks, French fries, and low-fat spread more frequently, and fish and fruits less frequently (*p* < 0.05) compared to parents in low-risk families. Paternal intake of vegetables and fish increased the odds for children being consumers of vegetables (OR 1.7; 95 % CI 1.0–2.9) and fish, respectively (OR 2.5; 95 % CI 1.4–4.4). Infant relative weight was weakly associated with a high intake of milk cereal drink (*r* = 0.15; *p* < 0.05), but not with any other aspect of dietary intake, obesity risk, or early feeding patterns.

**Conclusions:**

At the age of one, dietary intake in infants is not associated with family obesity risk, nor with parental obesogenic food intake. Milk cereal drink consumption but no other infant dietary marker reflects relative weight at this young age.

## Introduction

Many children develop obesity already during the preschool years [1], but little is known about which components of dietary intake are involved and at what age potential obesogenic dietary habits emerge [1, 2]. There is a need for more prospective studies to identify associations between dietary habits in young children and subsequent development of obesity [1]. The shaping of children’s food intake and preferences begins during the first year of life [3, 4], and parental food habits and home food availability are likely to exert an important impact [5].

Better knowledge about early associations between infant and parental dietary intake in relation to family obesity risk is needed to determine whether familial vulnerability for obesity would be expressed through dietary intake. However, whether dietary intake differs in young children at different risk of obesity is not clear. Some studies have identified differences in dietary intake and food preferences between preschool children with lean and obese parents [6–8], whereas others have not seen any differences in energy or fat intake, or energy density [9–11]. Other family socio-demographic factors, such as parental age, education, smoking status, and the presence of older siblings, also may affect the diet of preschool children [12–15]. Associations between food intake of toddlers and preschool children and of their mothers have been identified [12, 16–18], but the potential impact of the fathers’ diet on the intake of their offspring has been less studied [19]. However, few studies have compared the dietary intake in children at different obesity risk and analyzed associations to parental food habits as early as at age one [11, 12].

Associations between early infant feeding patterns (e.g., breast-feeding vs. bottle/formula feeding, timing of introduction of complementary foods) and weight development have been extensively studied [20, 21]. Notably, a high protein intake during the first years has been linked to later adiposity [22, 23]. However, associations between diet and weight at the age when the transition to family foods is initiated have not been fully explored [24], although there are indications that the diet of 1-year-old infants may be of higher quality compared to that of children 2–6 years old [25].

The aim of the present study was to compare infant and parental dietary intake between families with high and low obesity risk based on parental weight status, and to analyze associations between child and parental dietary intake. Additionally, we aimed to analyze associations between children’s relative weight and dietary intake, adjusting for obesity risk as defined by parental weight status and other factors with potential impact on child weight development such as socio-demographics and infant feeding. Parental adiposity, although a strong risk factor for offspring obesity, does not seem to affect the child’s weight as early as in infancy [11, 26, 27]. We therefore hypothesized that infants’ dietary intake would not yet be affected by obesity risk as measured by parental weight status, even though differences in parental food intake were expected.

## Materials and methods

### Study population: high- and low-risk families

This study is based on baseline data of the ongoing longitudinal obesity intervention Early Stockholm Obesity Prevention Project (Early STOPP), targeting children 1–6 years at high and low risk of obesity, based on parental weight status. The research protocol for the Early STOPP has been published elsewhere [28]. Ethical approval was given by the Stockholm Regional Ethical Review Board (2009/217-31).

Families were recruited to the Early STOPP based on parental body mass index (BMI); they were eligible for the high-risk group if at least one parent was obese (BMI ≥ 30) or both parents were overweight (BMI 25–29.9) and for the low-risk group if both parents were of normal weight (BMI < 25). In families with one obese parent, the other parent was either overweight or normal weight. Families with one overweight and one normal weight parent were not eligible for any of the groups. We henceforward refer to these two groups of families defined in this way based on parental weight status as high-risk and low-risk families. Written informed consent was obtained from the parents. In total, 240 children 12 months old (±2 months) were measured at the baseline visits, from January 2010 until June 2013. Parents were able to communicate in Swedish. For twin pairs, the firstborn twin was included. Exclusion criteria for the Early STOPP were chronic health problems likely to influence growth, physical activity, or eating habits. An additional inclusion criterion for the current study was that a three-day food record for the child had been completed by the parents (*n* = 193).

### Measures and procedures

#### Anthropometric data

Details of the baseline measurements have been reported elsewhere [29]. Measured weight and height were used to calculate BMI for children and parents and BMI standard deviation score (BMI SDS) for the children as indicators of relative weight. The children’s basal metabolic rate (BMR) was calculated according to the Shofield equation, recommended by the World Health Organization, using gender, weight, and height [30].

#### Demographic data

Questionnaires, one for each parent and one for the child, were completed by the parents. Data on duration of exclusive breast-feeding (<2 vs. ≥2 months), age at introduction of complementary foods, child care, and number of siblings as well as parental age, educational level, smoking habits, and ethnic origin were extracted. Parental educational level was combined into family education (low = both parents’ educational level <12 years).

#### Child dietary intake

The parents were instructed to complete an estimated food record during four consecutive days (two weekdays, two weekend days) for their child. Detailed instructions and booklets with pictures of portion sizes of typical meals and sizes of common foods were provided. For those children who had started kindergarten, similar information was provided to the personnel, who were asked to record the meals eaten at the kindergarten (kindergarten starts at age one in Sweden). Food records completed for at least 3 days were included in the analyses. Food records only comprising weekdays were included in the analyses, because no differences in nutritional content between records including weekend days and those including only weekdays were seen. For each day, the parent assessed whether it was a typical day and noted whether the child was sick. Atypical days and sick days were included in the analyses as assessed by the research team to be normal for children of this age. The quality of the food records (details of foods, brands, and estimation of food sizes) was assessed by the research team, and no food record was excluded due to low quality. The amount of breast milk was approximated using the time noted for breast-feeding and estimated amount per minute [31]. The energy and nutrient content was calculated using the Dietist XP software (version 3.2, Kost-och Näringsdata, Bromma, Sweden), which includes the Swedish national food database and supplier-delivered nutritional content of baby foods. For foods not available in the food database, alternative products very similar in energy and nutritional content were selected, and the fat content and quality were assured by adding small amounts of either butter or rapeseed oil. Dietary supplements were not included in the analyses. The children’s intake of macronutrients and different types of fat were compared to the Nordic Nutrition Recommendations, NNR 2012 [32].

Potential underreporting of the children’s energy intake was estimated by calculating the ratio of reported mean energy intake (EI) to BMR and comparing this ratio to the adapted Goldberg lower confidence interval cutoff [33]. References for physical activity levels and intra-subject variation in EI and BMR for young children were taken from Black [33]. The resulting BMR/EI ratio cutoffs were 0.85 for boys and 0.89 for girls.

Dichotomous diet quality indicators, representing potentially obesogenic (i.e., obesity-inducing) or obesity-protective habits and the degree of introduction to family meals, were determined based on whether the infants were consumers of predefined food items. The indicators were chosen based on being included in dietary indices or patterns used to study links between whole diet and health in young children [34], and representing dietary intake components of the Early STOPP intervention [28]. The children **w**ere defined to be a “consumer” of a food item if it occurred once or more times in the food record. Obesogenic indicators were “high-sugar foods,” “soft drinks,” and “salty snacks.” An additional potential obesogenic indicator was defined based on the amount of milk cereal drink (MCD) intake per day (>600 ml/day), based on evidence from previous Swedish studies showing a positive association between MCD consumption and relative weight in young children [35, 36]. MCD is an iron-fortified, follow-on formula based on cow’s milk and cereal, which is extensively used in Sweden from about 6 months of age. Obesity-protective indicators were “wholegrain bread or grains,” “fish,” “at least two different types of separate vegetables” (=during all days; vegetables as part of mixed dishes not included), “fruits daily” (=at 3 days), and “water at main meals” (=at all main meals).

#### Parental food intake

Both mothers and fathers completed a 17-item food frequency questionnaire (FFQ). The FFQ previously had been developed and validated by the Swedish National Food Agency [37]. Frequencies per day or week were calculated for selected foods (fruits and vegetables; whole grain bread; fish; French fries; sausage; full fat cheese; sweets and chocolates; buns, biscuits, and cakes; and soft drinks). The type of spread was defined as low fat (<40 % fat) or high fat (>60 %). The selected items had been associated previously with diet quality indicators for fat, saturated fat, sugar, and dietary fiber [37]. A food index was calculated based on these indicators, representing healthy food habits according to the Swedish dietary guidelines [38].

### Statistical analyses

Outcome variables were tested for normality with the Shapiro–Wilk test and through visual inspection of histograms. Nonparametric tests were used for variables not normally distributed.

The independent *t* test and *χ*^2^ test were used to test differences in child and parental characteristics between high- and low-risk families, and between study population and excluded families. The Mann–Whitney *U* test examined differences in child energy and macronutrient intake, and parental food frequencies between high- and low-risk families (independent groups). The *χ*^2^ test tested differences in child quality indicators by high/low obesity risk. The infant’s dietary intake was additionally compared between high- and low-risk families in multivariate analyses using linear regression, adjusting for gender, BMR, family socio-demographic factors (parental education, ethnicity, and smoking habits; the child being the firstborn child), early infant feeding (duration of exclusive breast-feeding, introduction of complementary foods), and breast-feeding status at age one.

The odds (OR) for infants’ diet quality indicators being high in relation to the corresponding parental diet quality indicators were determined using logistic regression, adjusting for high/low obesity risk, parental socio-demographic factors (education, ethnicity, smoking habits, firstborn status), child care (mother and/or father), and breast-feeding status at age one. Associations between children’s BMI SDS and dietary intake (energy, macronutrients, and diet quality) were analyzed in linear regression analyses, adjusting for high/low obesity risk, parental educational level, and early infant feeding (duration of exclusive breast-feeding, age at the introduction of complementary foods), breast-feeding status at age one and birth weight.

All tests were two-sided, and *p* values <0.05 were regarded as statistically significant. No correction for multiple testing using the Bonferroni method was applied, due to correlations between many of the tested variables (the Bonferroni method assumes totally independent tests and variables, and thus may be too conservative). In the interpretation of the results, the risk that one out of 20 significant results may be caused randomly was incorporated. All statistical analyses were performed using STATISTICA, version 11 (StatSoft, Inc., Tulsa, OK, USA, www.statsoft.com).

## Results

### Population characteristics

The study population (*n* = 193; Table 1) did not differ significantly compared to the excluded families due to no or incomplete child food records (*n* = 47) with regard to child and parental anthropometrics, parental weight status, parental educational level, or whether the child was firstborn (data not shown; *p* > 0.20). Of the high-risk families, 16 % had two overweight parents, in 24 % the father was obese, in 36 % the mother was obese, and in 24 % both parents were obese (data not shown). There were no significant differences in the children’s anthropometry at 12 months between high- and low-risk families (*p* > 0.05). Most children’s BMI SDS was within normal range, but two children had a BMI SDS above two and 10 had a BMI SDS below minus two; these 12 children were all in the high-risk group (data not shown). In the high-risk families, a higher proportion of the subjects had low family education (29 vs. 10 %), were breast-fed exclusively for <2 months (32 vs. 10 %), were the firstborn child (47 vs. 30 %), and had mothers who were trying to lose weight (53 vs. 35 %) compared to the low-risk families. Overall, only 3 % of the children had started to attend kindergarten; the mother was home with the child in 44 % of the families, the father in 22 %, and both parents in 30 %. In total, 16 % of the children were breast-fed at 12 months.

**Table 1:** Characteristics of the study population in high- and low-risk groups

	All *n* = 193		High risk (HR)^a^ *n* = 143		Low risk (LR)^a^ *n* = 50		*p* ^b^
	*n* (%)	Mean (SD)	*n* (%)	Mean (SD)	*n* (%)	Mean (SD)	
*Child characteristics*							
Age (years)^c^	192	1.00 (0.08)		0.99 (0.09)		1.02 (0.06)	0.07
Weight (kg)	191	10.2 (1.2)	142	10.2 (1.3)	49	10.1 (0.9)	0.79
Height (cm)	192	76.4 (3.0)	143	76.4 (3.0)	49	76.2 (2.9)	0.69
BMI (kg^2^/m)	191	17.4 (1.4)	142	17.4 (1.5)	49	17.5 (1.1)	0.90
BMI SDS	191	−0.45 (1.1)	142	−0.46 (1.1)	49	−0.40 (0.9)	0.70
BMR (kcal/day)	191	537 (46)	142	538 (48)	49	535 (41)	0.68
Sex, male	92 (48)		72 (50)		20 (40)		0.21
Firstborn	81 (42)		66 (47)		15 (30)		*
Breast-fed at 12 months	30 (16)		24 (17)		6 (12)		0.42
Breast-fed <2 months	50 (27)		45 (32)		5 (10)		**
Age at introd. complementary foods (months)	186	4.8 (1.0)	138	4.8 (1.0)	48	4.7 (0.8)	0.75
High risk (%)	74		100		0		
*Parental characteristics*							
Mother							
Age (years)		33.6 (4.5)	143	33.5 (4.5)	49	34.0 (4.8)	0.47
BMI (kg/m^2^)^d^		29.3 (6.8)	142	31.7 (6.3)	49	22.5 (2.2)	***
Trying to lose weight	92 (48)		75 (53)		17 (35)		*
Father							
Age (years)		35.7 (5.3)	133	35.5 (5.1)	48	36.5 (5.8)	0.27
BMI (kg/m^2^)		28.1 (5.1)	132	29.9 (4.7)	49	23.0 (1.5)	***
Trying to lose weight	52 (27)		49 (37)		3 (7)		^f^
*Socio-demographic factors*							
Parental education level^e^							
Mother low education	70 (36)		57 (40)		13 (26)		0.07
Father low education	80 (41)		68 (52)		12 (26)		**
Family low education	45 (24)		40 (29)		5 (10)		**
Ethnicity, other than Nordic							
Mother	19 (10)		13 (9)		6 (12)		^f^
Father	21 (11)		20 (14)		1 (2)		^f^
At least one parent	33 (17)		27 (19)		6 (12)		0.27
Smoking habits							
Mother smoker	10 (5)		7 (5)		3 (6)		^f^
Father smoker	13 (8)		8 (6)		5 (11)		^f^
At least one parent smoker	17 (9)		11 (8)		6 (12)		0.34
*Child care*							0.79
Mother	83 (44)		65 (46)		18 (38)		
Father	41 (22)		30 (21)		11 (23)		
Both parents	57 (30)		41 (29)		16 (34)		
Kindergarten	6 (3)		4 (3)		2 (4)		

^a^High risk = at least one parent obese or both parents overweight; low risk = both parents of normal weight

^b^ *p* value; difference between high- and low-risk groups; *t* test/*χ* ^2^ test. * *p* < 0.05; ** *p* < 0.01; *** *p* < 0.001

^c^Age at baseline measurement

^d^For pregnant women inclusion BMI is reported instead of baseline BMI (*n* = 10)

^e^Low education: <12 school years; Family low education: both parents have low education level

^f^Not determined; *χ* ^2^ test is not applicable, number of individuals <5 in some cells

### Child dietary intake

None of the children was identified as an underreporter of EI according to the calculated cutoff (see above). The mean EI/BMR ratio was 1.67. The mean intake of energy and macronutrients of the children adhered to the Nordic Nutrition Recommendations, except for the mean intake of energy-adjusted saturated fat (11 % of the EI; E%), which exceeded the recommended level of 10 E% (Table 2). The proportion of children following the recommendations for saturated fat (SFA) was 37 %. The proportion of children having a higher energy-adjusted protein intake compared to the recommendations (above 15 E%) was 21 %.

**Table 2:** Children’s dietary intake and diet quality indicators, in high- and low-risk groups

	NNR 2012 Children 12–23 months	%	m (SD)	All *n* = 193			%	m (SD)	High risk^a^ *n* = 143			%	m (SD)	Low risk^a^ *n* = 50			*p* ^b^
				Below rec. (%)	Follow rec. (%)	Above rec. (%)			Below rec. (%)	Follow rec. (%)	Above rec. (%)			Below rec. (%)	Follow rec. (%)	Above rec. (%)	
*Dietary intake*																	
Energy (kJ/kg)	337 kJ/kg bw (boys) 333 kJ/kg bw (girls)		368 (71)			68		364 (71)			65		378 (69)			76	0.25
Protein (E%)	10–15		13.4 (2.1)	3	76	21		13.4 (2.2)	5	72	23		13.5 (1.6)	0	86	14	0.60
Carbohydrates (E%)	45–60		52.5 (5.2)	5	89	6		52.5 (5.5)	6	88	6		52.7 (4.1)	4	90	6	0.98
Fat (E%)	30–40		32.5 (5.1)	28	65	7		32.8 (5.4)	28	64	8		31.9 (4.0)	30	68	2	0.46
SFA (E%)	<10		11.0 (2.6)			63		11.0 (2.7)			62		10.9 (2.4)			64	0.90
MUFA (E%)	–		13.1 (2.6)					13.2 (2.8)					12.8 (1.8)				0.48
PUFA (E%)	–		6.2 (1.3)					6.2 (1.5)					5.9 (0.9)				0.22
Dietary fiber (g/MJ)	–		2.6 (0.6)					2.6 (0.6)					2.8 (0.6)				0.06
*Diet quality indicators (consumers)*																	
Water to drink at all main meals		86					88					82					0.38
Fruits/berries daily		80					78					84					0.45
Vegetables (at least two different sorts)		47					45					52					0.38
Family meals/table foods		75					73					78					0.52
Whole meal bread/grains		35					36					32					0.58
Fish		65					65					64					0.90
Milk cereal drink > 600 ml/day		10					10					8					0.61
High-sugar foods		51					50					54					0.66
Sugar-sweetened beverages		11					13					8					0.38
Salted snacks		5					2					6					0.30

^a^High risk = at least one parent obese or both parents overweight; Low risk = both parents of normal weight

^b^ *p* value; difference in means in dietary intake and eating behaviors and differences in proportions in dichotomous diet quality indicators between high- and low-risk groups; Mann–Whitney *U* test/*χ* ^2^ test

No significant differences in EI or energy-adjusted intake of macronutrients, fat quality, or dietary fiber could be detected between high- and low-risk infants (Table 2). These results were confirmed in multivariate analyses, adjusting for gender, BMR, socio-demographics, early infant feeding, and breast-feeding at age one. The multivariate analyses additionally showed that the infants’ dietary intake was not associated with parental education (*p* > 0.05; data not shown). The relative intake of fat and saturated fat was positively associated with breast-feeding at age one (*p* < 0.05). Additionally, significant positive associations were identified between fat intake and fathers being of non-Nordic origin, and between saturated fat intake and smoking in mothers (*p* < 0.05; data not shown). Infant dietary intake was not associated with the child being firstborn or having siblings.

The proportion of the children adhering to the nutritional recommendations was the same in high- and low-risk families, with one exception: it was more common among the high-risk infants not to adhere to the recommendations for protein intake (28 vs. 14 %; *p* < 0.05), and there was a tendency for a larger proportion of the infants to have a protein intake above the recommended 10–15 E% (23 vs. 14 %; *p* = 0.17). The proportion of the infants with high quality for the different diet quality indicators was similar in high- and low-risk families.

### Parental food intake

The parents’ food intake differed for several obesogenic indicators between high- and low-risk families (Table 3). Mothers in high-risk families consumed fruit less often and soft drinks, French fries, and low-fat spread more often (*p* < 0.05). Fathers in high-risk families consumed fruit and fish less often and French fries more often (*p* < 0.05). There were no significant differences between the high- and low-risk parents in the total diet index score.

**Table 3:** Parents’ food intake in high- and low-risk groups

	All *n* = 193		High risk^a^ *n* = 143		Low risk^a^ *n* = 50		*p* ^b^
	%	Mean (SD)	%	Mean (SD)	%	Mean (SD)	
*Mothers’ food intake*							
Selected food frequencies (servings)							
Fruits, berries/day		1.4 (1.0)		1.3 (1.0)		1.7 (1.0)	*
Vegetables/day		1.5 (0.8)		1.5 (0.8)		1.5 (0.7)	0.60
Wholegrain bread slices/day		1.5 (1.4)		1.5 (1.3)		1.6 (1.6)	0.48
Fish, shellfish/week		1.3 (0.9)		1.2 (0.8)		1.5 (1.2)	0.20
Cheese 24–40 % fat/week		4.3 (3.8)		4.0 (3.1)		5.4 (5.3)	0.19
Sausage meals/week		1.0 (0.8)		1.0 (0.8)		0.9 (1.2)	0.11
French fries, fried potatoes/week		0.4 (0.5)		0.5 (0.6)		0.3 (0.3)	**
Sweets, chocolate/week		2.8 (2.5)		3.0 (2.6)		2.4 (2.3)	0.09
Pastries (cookies, cakes, buns/week		2.0 (2.1)		2.0 (2.2)		2.0 (1.7)	0.52
Sugar-sweetened beverages/week		2.4 (4.6)		2.9 (5.1)		0.9 (1.4)	**
Butter spread, low fat (≤40 %)	34		38		22		*
Diet index score (0–12 p)		5.1 (1.8)		4.9 (1.8)		5.4 (1.6)	0.14
*Fathers’ food intake*							
Selected food frequencies (servings)							
Fruits, berries/day		1.3 (1.5)		0.7 (0.7)		1.0 (0.6)	*
Vegetables/day		1.2 (0.7)		1.2 (0.7)		1.3 (0.7)	0.15
Wholegrain bread slices/day		1.3 (1.5)		1.3 (1.5)		1.5 (1.4)	0.23
Fish, shellfish/week		1.2 (1.0)		1.1 (0.8)		1.6 (1.2)	*
Cheese 24–40 % fat/week		4.0 (3.1)		3.7 (2.9)		4.8 (3.6)	0.09
Sausage meals/week		1.1 (1.3)		1.1 (1.3)		1.1 (1.3)	0.27
French fries, fried potatoes/week		0.8 (0.8)		0.8 (0.8)		0.7 (0.9)	*
Sweets, chocolate/week		2.1 (1.9)		2.1 (2.0)		2.0 (1.7)	0.64
Pastries (cookies, cakes, buns/week		2.0 (2.2)		1.9 (1.9)		2.2 (2.9)	0.99
Sugar-sweetened beverages/week		2.7 (4.5)		3.0 (4.9)		1.9 (2.6)	0.17
Butter spread, low fat (≤40 %)	32		36		21		0.06
Diet index score (0–12 p)		4.5 (1.7)		4.4 (1.6)		4.8 (1.6)	0.19

^a^High risk = at least one parent obese or both parents overweight; Low risk = both parents of normal weight

^b^ *p* value; difference between high- and low-risk groups; Mann–Whitney *U* test; * *p* < 0.05, ** *p* < 0.01

### Associations between child and parental diet quality indicators

The odds for children being consumers of at least two different types of vegetables were associated with the fathers’ intake of vegetables (adjusted OR 1.7, 95 % CI 1.0–2.9; adjusted for obesity risk, socio-demographics, infant feeding, breast-feeding status at age one, and child care; Table 4). There was an association between children being consumers of fish and fathers’ intake of fish (adjusted OR 2.5, 95 % CI 1.4–4.4). No other associations between the parental and child diet quality were identified. Obesity risk did not significantly affect these associations.

**Table 4:** Associations between child and parental diet quality: adjusted odds ratios (OR)^a^ for child quality indicators in relation to parental food frequencies

Parental food frequencies	Child diet quality indicator											
	Fruits/berries daily		Two discrete vegetables		Whole meal bread/grains		Fish meals		Sweets introduced		Sugar-sweetened beverages	
	OR	95 % CI	OR	95 % CI	OR	95 % CI	OR	95 % CI	OR	95 % CI	OR	95 % CI
Fruits, berries/day—mother	1.2	0.7–1.9										
Fruits, berries/day—father	2.0	0.8–4.6										
Vegetables/day—mother			1.2	0.7–1.9								
Vegetables/day—father			1.8	1.0–3.0*								
Wholegrain bread slices/day—mother					0.9	0.7–1.3						
Wholegrain bread slices/day—father					1.2	0.9–1.6						
Fish, shellfish/week—mother							1.0	0.6–1.7				
Fish, shellfish/week—father							2.4	1.4–4.4*				
Sweets and pastries^b^/week—mother									1.0	1.0–1.1		
Sweets and pastries^b^/week—father									1.0	0.9–1.0		
Sugar-sweetened beverages/week—mother											1.1	1.0–1.2
Sugar-sweetened beverages/week—father											0.9	0.7–1.1

* *p* < 0.05

^a^Odds ratios adjusted for obesity risk, socio-demographics (parental education, non-Nordic background, smoking status, firstborn), breast-feeding status at age one, and child care

^b^Includes sweets, chocolates, cookies, cakes, and buns

### Associations between child dietary intake and weight

The infants’ BMI SDS at age one was not associated with energy or macronutrient intake, obesity risk, or infant feeding (Table 5). Regarding the diet quality indicators, only a high intake of MCD (>600 ml/day) was associated with BMI SDS (*r* = 0.15, *p* < 0.05), but this association became nonsignificant in multivariate analyses including birth weight (*β* = 0.11; *p* = 0.17). The absolute intake of energy, protein, carbohydrates, fat, dietary fiber, and MCD correlated positively to BMR (*r* = 0.15–0.32; *p* < 0.05).

**Table 5:** Associations between children’s energy-adjusted dietary intake and BMI SDS, and between absolute dietary intake and BMR (basal metabolic rate)

	Associations with BMI SDS			Associations with BMR	
	Unit	Unadjusted	Adjusted^b^	Unit	*r* ^a^
		*r* ^a^	*β*		
*Dietary intake per day*					
Energy	kJ	0.03	0.06	kJ	0.33***
Protein	E%	0.02	0.03	g	0.30***
Carbohydrates	E%	0.00	0.01	g	0.30***
Fat	E%	0.03	−0.03	g	0.25**
Dietary fiber	g/MJ	0.03	0.05	g	0.19**
Milk cereal drink	>600 ml	0.15*	0.11	ml	0.15*

* *p* < 0.05; ** *p* < 0.01; *** *p* < 0.001

^a^Spearman’s rank correlation coefficient

^b^Adjusted for obesity risk as determined by parental weight status, parental education, early infant feeding, breast-feeding status at age one, and birth weight

## Discussion

This study demonstrates that the dietary intake of 1-year-old infants is not associated with family obesity risk as assessed by parental weight status in a high-income country. Even though an obesogenic dietary pattern was apparent among the parents in the high-risk families, their reported intake of unhealthy foods was not associated with the children’s diet quality at this early age. Furthermore, the children’s relative weight was only weakly associated with a high intake of MCD, but not with any other aspect of dietary intake, nor with obesity risk based on parental weight status.

Our hypothesis regarding a lack of influence of obesity risk and obesogenic parental food intake on infants’ dietary intake and relative weight was thus confirmed. Our results are in agreement with an earlier study in a small sample of high- and low-risk US infants [11]. Also, in a recent study of infant feeding patterns during the first year, no significant impact of parental adiposity was identified [39]. Most other research showing differences in dietary intake between children at high and low risk of obesity has involved children older than 1 year [6–8, 40]. In another study on the Early STOPP infants, the lack of impact of parental adiposity on infant growth during the first year has been discussed in detail [29].

Regarding the impact of the obesogenic parental food habits, it is likely that children at age one have not yet been heavily exposed. As shown, about half of the children had been exposed to high-sugar foods, and only one in 10 had been introduced to sugar-sweetened beverages. While family foods have been introduced to most children at age one, typical infant foods such as infant formula, MCD and porridge, and ready-made baby dishes still constitute a large part of the diet, moderating the impact of the parental food habits [25, 41]. There is evidence that the food intake in 1-year-old infants differs compared to that of children 2–6 years old, and that young children’s diet quality gradually decreases by age [25, 42, 43]. Also, as parent–child resemblance in unhealthy food intake has been seen to be stronger among older children [44], it is plausible that the infant’s dietary intake will become more influenced by unhealthy parental food intake as they grow older. In addition to the parental influence, having older siblings may also affect children’s dietary intake [45]; however, no significant association was seen at this age.

The identified positive associations between paternal and child intake of several healthy foods (vegetables and fish) are noteworthy. The mother’s and child’s diets have been linked in many studies [12, 16–18, 46], although the resemblances are relatively weak [44]. However, recently correlations between paternal and child diet intake have also been identified in an Australian sample of primary-school-aged children [19]. These findings suggest that the parental influence on the child’s food intake at this age mainly acts through role modelling of food preferences and the availability to foods that parents bring into the home [47]. In comparison, in a large Swedish cohort, the intake of high-sugar foods in 1-year-old children was associated with maternal intake of high-sugar foods during pregnancy [12], where a possible mechanism instead may be through influencing the child’s taste preferences during pregnancy and lactation [48]. Child care and parental leave systems are likely to influence parent’s ability to role model food intake early in infancy. In Sweden, children do not attend kindergarten before the age of one, and a substantial proportion of fathers’ is involved in the daily care of their child at this age. The identified associations between paternal and child diet in our study may reflect that more than half of the fathers were involved in the daily care of the child. It is not clear why no associations with the mother’s food intake were identified, but one potential explanation is that more mothers than fathers were trying to lose weight and thus trying to keep to a diet not appropriate for their child.

No clear pattern between child diet and parental socio-demographic factors could be identified. Although several other studies on infants’ dietary intake have identified an impact of socio-demographic factors, such as maternal education, age, and smoking [12–15], these associations may become progressively stronger as the children get older [49, 50]. In a Finnish sample of preschool children, a low dietary quality was not associated with socio-demographic factors as early as at age one, possibly due to a low variation in the diet because of the high consumption of commercial baby foods at this age, similar to the findings of our study [50]. On the other hand, parental education has been associated with early infant feeding (breast-feeding duration) [51], although this association could not be seen in our previous study in the Early STOPP infants [29]. It should be noted that all Swedish infants visit their child healthcare center frequently during the first year, and parents receive detailed information on when and how to introduce complementary foods. Thus, adequate knowledge of and adherence to nutrition recommendations among the parents is also a plausible explanation for socio-demographics not affecting the infants’ dietary intake in our population.

We did not identify any associations between the infants’ intake of energy and macronutrients and their relative weight (BMI SDS) at age one. The absence of an association is not likely to be explained by implausible reporting, as absolute intake of macronutrients correlated positively to weight and basal energy requirements. It is possible that the variation in infants’ relative weight is explained to a greater extent by energy expenditure [52]; however, this was not measured in this study. In contrast to our results, cross-sectional associations between EI and BMI z-score have been identified in children 2–9 years old reporting plausible EIs [53]. There is also evidence of longitudinal associations between EI during infancy and higher BMI in childhood [11, 24], which may indicate that EI predicts weight gain, whereas weight only marginally predicts EI [11].

The association between MCD consumption and BMI SDS confirms two recently published Swedish studies. In a large cohort of infants, MCD intake at 6 months increased the risk of a high BMI at 12 and 18 months [35]. A longitudinal study on the Swedish cohort of the IDEFICS study showed that MCD consumers were nearly five times more likely to develop overweight [36]. In contrast to these studies, we could not confirm that MCD consumption was associated with parental obesity or educational level. The effect of MCD on relative weight became nonsignificant when birth weight was added to the model; however, it should be acknowledged that birth weight has a very strong impact on relative weight of infants at age one as shown by our own previous study [26]. MCD is a very typical and common Swedish infant product, and many Swedish children continue to consume MCD after infancy, even after toddlerhood. Also, MCD is easily ingested in large volumes as it is bottle-fed. This suggests that the effect of MCD consumption on Swedish children’s weight development should be further studied prospectively.

Early infant feeding patterns (short duration of exclusive breast-feeding, early introduction of complementary foods) did not have an impact on the children’s dietary intake in our study, although these associations have been seen in other studies [12, 39, 54, 55]. Neither was early infant feeding associated with BMI SDS at age one, in contrast to previous research that indicated that formula may have a growth-accelerating effect compared to breast-feeding [56, 57]. However, similar to our study, another Swedish study failed to identify associations between infant feeding patterns and relative weight up to 18 months [58]. On the other hand, a tendency for the high-risk children to have a high protein intake compared to nutritional recommendations was observed. This may increase the risk for subsequent adiposity, because longitudinal studies have shown that a high protein intake at age one and 2 years independently is associated with a higher relative weight and risk for overweight later in childhood [22, 23, 59].

Our analyses support a valid reporting of infant dietary intake, as it correlated to basal energy requirements and no underreporting of EI could be identified. Also, the reported intake of energy and fat was comparable to a previous study of Swedish infants, where a too-high intake of saturated fat according to the recommendations was reported just as in our study [60]. It has been described before that parents reasonably accurately report the food intake of their infants [61]. The assessment of the validity of reported food intake should preferably additionally be based on objectively measured biomarkers for food intake, such as fatty acid composition in blood. These analyses will be performed in future studies on this material.

The strength of this study is the design where dietary intake in high-risk and low-risk infants is compared, testing whether the familial vulnerability for obesity would be expressed through food intake. Additionally, anthropometrics were measured in both children and parents. Also, potential underreporting of infant EI was tested using the adapted Goldberg method. Finally, many of previously suggested potential determinants for infant dietary intake and weight development were included as covariates in the study.

Some limitations of the study should be acknowledged. Residual confounding is possible; there may be differences between the high- and low-risk groups that were not measured and adjusted for. Furthermore, the size of the population may limit the possibility to detect statistically significant differences in dietary intake and the number of factors possible to adjust for. Also, self-selection bias may have contributed to the nonsignificant results, as the Early STOPP parents are likely to be more interested in healthy dietary habits and more motivated to serve their child healthy foods than the source populations of high- and low-risk families.

In conclusion, at age one, infant dietary intake was not associated with family obesity risk, nor with parental intake of unhealthy foods. MCD but no other aspect of infant dietary intake reflects relative weight at this age.

## References

[1] van Stralen MM, te Velde SJ, van Nassau F, Brug J, Grammatikaki E, Maes L (2012). Weight status of European preschool children and associations with family demographics and energy balance-related behaviours: a pooled analysis of six European studies. Obes Rev.

[2] Moreno LA, Rodriguez G (2007). Dietary risk factors for development of childhood obesity. Curr Opin Clin Nutr Metab Care.

[3] Harris G (2008). Development of taste and food preferences in children. Curr Opin Clin Nutr Metab Care.

[4] Kral TV, Rauh EM (2010). Eating behaviors of children in the context of their family environment. Physiol Behav.

[5] Campbell KJ, Crawford DA, Ball K (2006). Family food environment and dietary behaviors likely to promote fatness in 5–6 year-old children. Int J Obes (Lond).

[6] Kral TV, Stunkard AJ, Berkowitz RI, Stallings VA, Moore RH, Faith MS (2008). Beverage consumption patterns of children born at different risk of obesity. Obesity (Silver Spring).

[7] Nguyen VT, Larson DE, Johnson RK, Goran MI (1996). Fat intake and adiposity in children of lean and obese parents. Am J Clin Nutr.

[8] Wardle J, Guthrie C, Sanderson S, Birch L, Plomin R (2001). Food and activity preferences in children of lean and obese parents. Int J Obes Relat Metab Disord.

[9] Kral TV, Stunkard AJ, Berkowitz RI, Stallings VA, Brown DD, Faith MS (2007). Daily food intake in relation to dietary energy density in the free-living environment: a prospective analysis of children born at different risk of obesity. Am J Clin Nutr.

[10] McGloin AF, Livingstone MB, Greene LC, Webb SE, Gibson JM, Jebb SA (2002). Energy and fat intake in obese and lean children at varying risk of obesity. Int J Obes Relat Metab Disord.

[11] Stunkard AJ, Berkowitz RI, Schoeller D, Maislin G, Stallings VA (2004). Predictors of body size in the first 2 y of life: a high-risk study of human obesity. Int J Obes Relat Metab Disord.

[12] Brekke HK, van Odijk J, Ludvigsson J (2007). Predictors and dietary consequences of frequent intake of high-sugar, low-nutrient foods in 1-year-old children participating in the ABIS study. Br J Nutr.

[13] Fernandez-Alvira JM, Mouratidou T, Bammann K, Hebestreit A, Barba G, Sieri S (2013). Parental education and frequency of food consumption in European children: the IDEFICS study. Public Health Nutr.

[14] Rogers I, Emmett P, Team AS (2003). The effect of maternal smoking status, educational level and age on food and nutrient intakes in preschool children: results from the Avon Longitudinal Study of Parents and Children. Eur J Clin Nutr.

[15] Smithers LG, Brazionis L, Golley RK, Mittinty MN, Northstone K, Emmett P (2012). Associations between dietary patterns at 6 and 15 months of age and sociodemographic factors. Eur J Clin Nutr.

[16] Greenberg RS, Ariza AJ, Binns HJ (2010). Activity and dietary habits of mothers and children: close ties. Clin Pediatr (Phila).

[17] Lee SY, Hoerr SL, Schiffman RF (2005). Screening for infants’ and toddlers’ dietary quality through maternal diet. MCN Am J Matern Child Nurs.

[18] McGowan L, Croker H, Wardle J, Cooke LJ (2012). Environmental and individual determinants of core and non-core food and drink intake in preschool-aged children in the United Kingdom. Eur J Clin Nutr.

[19] Hall L, Collins CE, Morgan PJ, Burrows TL, Lubans DR, Callister R (2011). Children’ intake of fruit and selected energy-dense nutrient-poor foods is associated with fathers’ intake. J Am Diet Assoc.

[20] Beyerlein A, von Kries R (2011). Breastfeeding and body composition in children: will there ever be conclusive empirical evidence for a protective effect against overweight?. Am J Clin Nutr.

[21] Pearce J, Taylor MA, Langley-Evans SC (2013). Timing of the introduction of complementary feeding and risk of childhood obesity: a systematic review. Int J Obes (Lond).

[22] Gunther AL, Buyken AE, Kroke A (2007). Protein intake during the period of complementary feeding and early childhood and the association with body mass index and percentage body fat at 7 y of age. Am J Clin Nutr.

[23] Rolland-Cachera MF, Deheeger M, Akrout M, Bellisle F (1995). Influence of macronutrients on adiposity development: a follow up study of nutrition and growth from 10 months to 8 years of age. Int J Obes Relat Metab Disord.

[24] Pearce J, Langley-Evans SC (2013). The types of food introduced during complementary feeding and risk of childhood obesity: a systematic review. Int J Obes (Lond).

[25] Kyttala P, Erkkola M, Kronberg-Kippila C, Tapanainen H, Veijola R, Simell O (2010). Food consumption and nutrient intake in Finnish 1-6-year-old children. Public Health Nutr.

[26] Svensson V, Ek A, Forssen M, Ekbom K, Cao Y, Ebrahim M (2014). Infant growth is associated with parental education but not with parental adiposity—Early Stockholm Obesity Prevention Project. Acta Paediatr.

[27] Svensson V, Jacobsson JA, Fredriksson R, Danielsson P, Sobko T, Schioth HB (2011). Associations between severity of obesity in childhood and adolescence, obesity onset and parental BMI: a longitudinal cohort study. Int J Obes (Lond).

[28] Sobko T, Svensson V, Ek A, Ekstedt M, Karlsson H, Johansson E (2011). A randomised controlled trial for overweight and obese parents to prevent childhood obesity—Early STOPP (STockholm Obesity Prevention Program). BMC Public Health.

[29] Svensson V, Ek A, Forssen M, Ekbom K, Cao Y, Ebrahim M (2014). Infant growth is associated with parental education but not with parental adiposity—Early Stockholm Obesity Prevention Project. Acta Paediatr.

[30] Schofield WN (1985). Predicting basal metabolic rate, new standards and review of previous work. Hum Nutr Clin Nutr.

[31] Paul AABA, Evans J, Cole TJ, Whitehead RG (1988). Breastmilk intake and growth in infants from two to ten months. J Hum Nutr Diet.

[32] Ministers NCo (2013) NNR 2012. Nordic Nutrition Recommendations 2012. Part 1 summary, principles and use. ISBN 978-92-893-2629-2

[33] Black AE (2000). Critical evaluation of energy intake using the Goldberg cut-off for energy intake:basal metabolic rate. A practical guide to its calculation, use and limitations. Int J Obes Relat Metab Disord.

[34] Smithers LG, Golley RK, Brazionis L, Lynch JW (2011). Characterizing whole diets of young children from developed countries and the association between diet and health: a systematic review. Nutr Rev.

[35] Almquist-Tangen G, Dahlgren J, Roswall J, Bergman S, Alm B (2013). Milk cereal drink increases BMI risk at 12 and 18 months, but formula does not. Acta Paediatr.

[36] Wiberger M, Eiben G, Lissner L, Mehlig K, Papoutsou S, Hunsberger M (2014). Children consuming milk cereal drink are at increased risk for overweight: the IDEFICS Sweden study, on behalf of the IDEFICS Consortium. Scand J Public Health.

[37] Sepp H, Ekelund U, Becker W (2004) Questionnaire on diet and physical activity in adults. Swedish National Food Agency Report 21. Swedish National Food Agency

[38] Becker W (2009) Indicators of healthy food habits. Results from interview surveys 2008. Swedish National Food Agency Report 22. Swedish National Food Agency

[39] Betoko A, Charles MA, Hankard R, Forhan A, Bonet M, Saurel-Cubizolles MJ (2013). Infant feeding patterns over the first year of life: influence of family characteristics. Eur J Clin Nutr.

[40] Pei Z, Flexeder C, Fuertes E, Standl M, Berdel D, von Berg A (2014). Mother’ body mass index and food intake in school-aged children: results of the GINIplus and the LISAplus studies. Eur J Clin Nutr.

[41] Briefel RR, Reidy K, Karwe V, Jankowski L, Hendricks K (2004). Toddlers’ transition to table foods: impact on nutrient intakes and food patterns. J Am Diet Assoc.

[42] Kudlova E, Schneidrova D (2012). Dietary patterns and their changes in early childhood. Cent Eur J Public Health.

[43] Lioret S, McNaughton SA, Spence AC, Crawford D, Campbell KJ (2013). Tracking of dietary intakes in early childhood: the Melbourne InFANT Program. Eur J Clin Nutr.

[44] Beydoun MA, Wang Y (2009). Parent–child dietary intake resemblance in the United States: evidence from a large representative survey. Soc Sci Med.

[45] Schrempft S, van Jaarsveld CH, Fisher A, Wardle J (2013). Family and infant characteristics associated with timing of core and non-core food introduction in early childhood. Eur J Clin Nutr.

[46] Robinson S, Marriott L, Poole J, Crozier S, Borland S, Lawrence W (2007). Dietary patterns in infancy: the importance of maternal and family influences on feeding practice. Br J Nutr.

[47] Vereecken C, Haerens L, De Bourdeaudhuij I, Maes L (2010). The relationship between children’s home food environment and dietary patterns in childhood and adolescence. Public Health Nutr.

[48] Mennella JA, Jagnow CP, Beauchamp GK (2001). Prenatal and postnatal flavor learning by human infants. Pediatrics.

[49] Cameron AJ, Ball K, Pearson N, Lioret S, Crawford DA, Campbell K (2012). Socioeconomic variation in diet and activity-related behaviours of Australian children and adolescents aged 2–16 years. Pediatr Obes.

[50] Kyttala P, Erkkola M, Lehtinen-Jacks S, Ovaskainen ML, Uusitalo L, Veijola R (2014). Finnish Children Healthy Eating Index (FCHEI) and its associations with family and child characteristics in pre-school children. Public Health Nutr.

[51] Wijlaars LP, Johnson L, van Jaarsveld CH, Wardle J (2011). Socioeconomic status and weight gain in early infancy. Int J Obes (Lond).

[52] Bleich SN, Ku R, Wang YC (2011). Relative contribution of energy intake and energy expenditure to childhood obesity: a review of the literature and directions for future research. Int J Obes (Lond).

[53] Hebestreit A, Bornhorst C, Barba G, Siani A, Huybrechts I, Tognon G (2014). Associations between energy intake, daily food intake and energy density of foods and BMI z-score in 2–9-year-old European children. Eur J Nutr.

[54] de Lauzon-Guillain B, Jones L, Oliveira A, Moschonis G, Betoko A, Lopes C (2013). The influence of early feeding practices on fruit and vegetable intake among preschool children in 4 European birth cohorts. Am J Clin Nutr.

[55] Gunnarsdottir I, Schack-Nielsen L, Michaelsen KF, Sorensen TI, Thorsdottir I, NordNet Study G (2010). Infant weight gain, duration of exclusive breast-feeding and childhood BMI—two similar follow-up cohorts. Public Health Nutr.

[56] Emmett PM, Jones LR (2014). Diet and growth in infancy: relationship to socioeconomic background and to health and development in the Avon Longitudinal Study of Parents and Children. Nutr Rev.

[57] Kramer MS, Guo T, Platt RW, Vanilovich I, Sevkovskaya Z, Dzikovich I (2004). Feeding effects on growth during infancy. J Pediatr.

[58] Almqvist-Tangen G, Dahlgren J, Roswall J, Bergman S, Alm B (2013). Milk cereal drink increases BMI risk at 12 and 18 months, but formula does not. Acta Paediatr.

[59] Scaglioni S, Agostoni C, Notaris RD, Radaelli G, Radice N, Valenti M (2000). Early macronutrient intake and overweight at five years of age. Int J Obes Relat Metab Disord.

[60] Ohlund I, Hornell A, Lind T, Hernell O (2008). Dietary fat in infancy should be more focused on quality than on quantity. Eur J Clin Nutr.

[61] Uusitalo L, Nevalainen J, Salminen I, Ovaskainen ML, Kronberg-Kippila C, Ahonen S (2013). Fatty acids in serum and diet—a canonical correlation analysis among toddlers. Matern Child Nutr.

